# The Mouse Estrus and Circadian Cycles Interact to Influence Behavioral Rhythms: Relevance to the Menstrual Cycle in Humans

**DOI:** 10.1177/07487304251321021

**Published:** 2025-02-27

**Authors:** Maya Purday

**Affiliations:** Department of Pharmacology, University of Oxford, Oxford, UK

The interaction between circadian rhythms and reproductive cycles is an important area in neuroscience and circadian biology. Alvord and Pendergast (2024) enhance our understanding of this interaction by demonstrating that the estrus cycle of female mice regulates the amplitude of circadian eating behavior. Their findings build on previous research by providing insight into how the fluctuations of endogenous estrogens modulate circadian-regulated behaviors beyond reproduction. This commentary explores what was learned beyond existing knowledge and how this may propel the field forward in the understanding of sex-based circadian differences.

The study found that female mice’s estrus cycle regulates eating behavior, with the highest amplitude of circadian eating during proestrus and estrus when estrogen levels are the highest. Experimenters watched recordings of the mice in their home cages to count instances of eating, then separated this into 10-minute intervals to show at which times of day they were eating the most. A high amplitude of eating is therefore when the day’s food intake is concentrated in a short time. Activity rhythm was measured by the number of times the wheel turned daily and associated with estrus stage by observing cells from a vaginal swab each day under the microscope. The authors found that the mice are most active during proestrus, coinciding with the highest eating amplitude. Importantly, these fluctuations persisted in constant darkness, showing that the behavioral rhythms are not driven by external light but by internal cues, such as hormonal and circadian cycles. This finding is significant as it extends our understanding of the intersection of reproductive and circadian cycles.

The authors present the perspective that the circadian cycle allows the coordination of behavior and physiology for increased reproductive success. Evidence of circadian disruption decreasing reproductive success by causing abnormal mating behavior and ovulation timing (Takasu et al., 2015) supports this. Previous work shows that exogenous estradiol impacts eating patterns (Omotola et al., 2019), but the present study is the first demonstration that endogenous estrogens regulate the strength of eating rhythms. Higher eating amplitude may allow female mice to maximize the time available for mating during their most fertile window by consuming more food in a short amount of time. Moreover, the authors show that ovariectomy, the complete removal of the ovaries and therefore the primary source of estrogens, disrupts the regular 4- to 5-day eating cycle. Taken together, these results underscore the essential role of ovarian hormones in maintaining circadian regularity in behavior.

One of the most important contributions from Alvord and Pendergast’s work is the emphasis on sex-specific circadian regulation. It has been found that estrogens can influence genes involved in the molecular circadian clock by binding to estrogen response elements (EREs) in DNA (Rossetti et al., 2012). The exact neural circuits involved in feeding behavior are unknown, but the suggestion that estradiol may regulate circadian clock genes in such brain regions raises the exciting prospect that the mechanism of behavioral regulation by endogenous estrogens may be investigated. Inhibiting their action at EREs in a brain region–specific manner with pharmacological or genetic tools in female mice may increase our understanding of how circadian and reproductive systems are integrated.

Traditionally, most circadian research has been conducted on male animals, partly to avoid the variation in data that may be associated with female reproductive cycles. Alvord and Pendergast demonstrate that these hormonal cycles are not mere confounding variables but play a critical role in regulating circadian behavior. These results reinforce the need for circadian studies to explicitly include females to better understand the sex differences in circadian regulation. Not accounting for these differences may lead to misinterpretation of data and hinder understanding of circadian disruption caused by hormonal changes.

Alvord and Pendergast’s demonstration of the link between the estrus cycle and circadian eating behavior may help understand impairments in metabolic health in patients with disrupted reproductive health. Disruption in hormone cycles has also been associated with symptoms characteristic of circadian disruption, including sleep disturbance and metabolic syndrome, such as in menopause (Kravitz et al., 2018). Ovariectomized females’ abnormal eating patterns in this study provide an avenue to test the impact of supplemental estrogen directly on regulating circadian behavioral disruption. Understanding how estrogens stabilize circadian rhythms could support hormone replacement therapy as a treatment for circadian-related disruption in menopause and other reproductive disorders, improving both reproductive and metabolic health.

While the study provides a solid foundation for understanding the hormonal regulation of circadian rhythms, several questions remain unanswered. This includes the exact role of estrogens’ influence on circadian clock gene expression in brain circuits governing eating behavior. The hypothalamus has long been implicated in the circadian control of eating—early studies show bilateral lesions to the rat hypothalamus abolish reliable 24-hour drinking rhythms (Stephan and Zucker, 1972). Future studies could use gene-editing tools to target estrogen receptors or clock genes in specific hypothalamic regions to yield deeper insights into the neural circuitry involved in circadian and hormonal regulation.

The estrus cycle in mice offers a useful model for understanding the fundamental biology of how hormones impact behavior but it has key differences with the menstrual cycle in humans. Translating these findings to humans will be informative in understanding the behavioral and metabolic consequences of how menstrual cycles affect circadian rhythms. The variations in sleep, a behavioral rhythm strongly regulated by the circadian cycle, across the menstrual cycle and its differences in women with reproductive disorders have been studied (Moderie et al., 2021). The method of having women with and without reproductive disorders come into the lab for monitoring every third day of their menstrual cycle could be adapted to include measurements such as daily rhythms in food intake and blood glucose levels to investigate how the menstrual and circadian cycles affect metabolism. Including groups of perimenopausal women who are and are not undergoing hormone replacement therapy will help to understand the effects of targeting reproductive health on circadian rhythms in eating behavior and their metabolic outcomes.
